# Nanomechanical Pyrolytic Carbon Resonators: Novel Fabrication Method and Characterization of Mechanical Properties

**DOI:** 10.3390/s16071097

**Published:** 2016-07-15

**Authors:** Maksymilian Kurek, Frederik K. Larsen, Peter E. Larsen, Silvan Schmid, Anja Boisen, Stephan S. Keller

**Affiliations:** 1 Department of Micro- and Nanotechnology, Technical University of Denmark, Kgs. Lyngby 2800, Denmark; frederikklarsen@gmail.com (F.K.L.); peem@nanotech.dtu.dk (P.E.L.); silvan.schmid@tuwien.ac.at (S.S.); Anja.Boisen@nanotech.dtu.dk (A.B.); Stephan.Keller@nanotech.dtu.dk (S.S.K.); 2 Institute of Sensor and Actuator Systems, TU Wien, Vienna 1040, Austria

**Keywords:** MEMS, resonators, pyrolysis, pyrolytic carbon, microfabrication

## Abstract

Micro- and nanomechanical string resonators, which essentially are highly stressed bridges, are of particular interest for micro- and nanomechanical sensing because they exhibit resonant behavior with exceptionally high quality factors. Here, we fabricated and characterized nanomechanical pyrolytic carbon resonators (strings and cantilevers) obtained through pyrolysis of photoresist precursors. The developed fabrication process consists of only three processing steps: photolithography, dry etching and pyrolysis. Two different fabrication strategies with two different photoresists, namely SU-8 2005 (negative) and AZ 5214e (positive), were compared. The resonant behavior of the pyrolytic resonators was characterized at room temperature and in high vacuum using a laser Doppler vibrometer. The experimental data was used to estimate the Young’s modulus of pyrolytic carbon and the tensile stress in the string resonators. The Young’s moduli were calculated to be 74 ± 8 GPa with SU-8 and 115 ± 8 GPa with AZ 5214e as the precursor. The tensile stress in the string resonators was 33 ± 7 MPa with AZ 5214e as the precursor. The string resonators displayed maximal quality factor values of up to 3000 for 525-µm-long structures.

## 1. Introduction

The dynamic development in the field of micro- and nanofabrication allowed the definition of a class of ultrasensitive micro- and nanomechanical sensors capable of detecting various physical variables [1]. The high sensitivity of these sensors is a great opportunity, in particular for mass [2], force [3] and thermal [4] sensing for numerous applications such as mass spectrometry [5], cell detection [6] and infra-red (IR) spectroscopy [7]. These devices typically consist of simple micromechanical structures such as singly-clamped cantilever beams or doubly-clamped bridges that exhibit resonant behavior. The principle of operation is generally based on the monitoring of the shift of the resonance frequency of the beams due to external factors such as the addition of mass or a change of temperature.

Micro- and nanomechanical resonators are typically fabricated from low-loss semiconductor materials and ceramics, such as silicon, silicon nitride, silicon carbide or aluminum nitride. The structuring of these materials requires a photolithography step and a chemical or physical etch. For the realization of micro- and nanoelectromechanical systems, the micromechanical resonators should preferentially be electrically conductive, which requires additional doping in the case of semiconductors and metallization in the case of ceramics.

In this work, we investigate pyrolytic carbon as a material for the fabrication of nanomechanical resonators. Pyrolytic carbon is conductive and can be obtained directly from photoresist through pyrolysis at elevated temperatures in inert atmosphere. The advantage of this fabrication method is that the geometry of carbon micro- and nanostructures such as micropillar arrays [8], bridges [9] and suspended nanowires [10] can be defined through simple photolithographic processes followed by pyrolysis. A few different approaches to fabricate carbon-based doubly-clamped beams [11] and cantilevers [12] were presented using soft lithography or multiple steps of photolithography. Here, our goal was (i) to demonstrate a simple and reliable fabrication process for pyrolytic carbon strings and cantilevers in only three processing steps consisting of photolithography, dry etching and pyrolysis and (ii) to explore the mechanical properties of the resulting structures in order to investigate the influence of processing conditions.

Pyrolytic carbon is a highly amorphous material and considerable friction loss could be expected compared to crystalline materials such as silicon. However, tensile stress can increase the quality factor (Q) of resonating structures significantly, as it was shown for string resonators made of silicon nitride [13,14] and SU-8 photoresist [15]. The tensile stress increases the energy stored in a resonator, which in essence decreases the effect of energy loss on the resulting Q [16,17], and hence the effect is known as damping dilution. During pyrolysis, considerable tensile stress is generated in pyrolytic carbon microstructures due to shrinkage and mass loss of the photoresist [18]. Hence, it should be possible to fabricate high-Q nanomechanical pyrolytic carbon string resonators based on the stress-induced damping dilution effect.

We fabricated pyrolytic carbon strings and cantilevers with two different photoresist precursors: SU-8 2005 (SU-8) and AZ 5214e (AZ). The resonant behavior of the microresonators was characterized and the results were used to estimate the Young’s moduli of pyrolytic carbon and the tensile stress in the string resonators.

## 2. Materials and Methods

### 2.1. Fabrication

The photolithographic mask design included singly- and doubly-clamped beams with different lengths *L* = 100–1000 µm and widths *w* = 3–50 µm. The fabrication process consisted of the three main steps: photolithography, dry etching and pyrolysis. Two different fabrication strategies were investigated, where the last two process steps were carried out in inverse order. After the photolithography either dry etching or pyrolysis was performed first followed by pyrolysis or dry etching, respectively. The process conditions of photolithography, dry etching and pyrolysis were identical regardless of the fabrication strategy.

**Figure 1:**
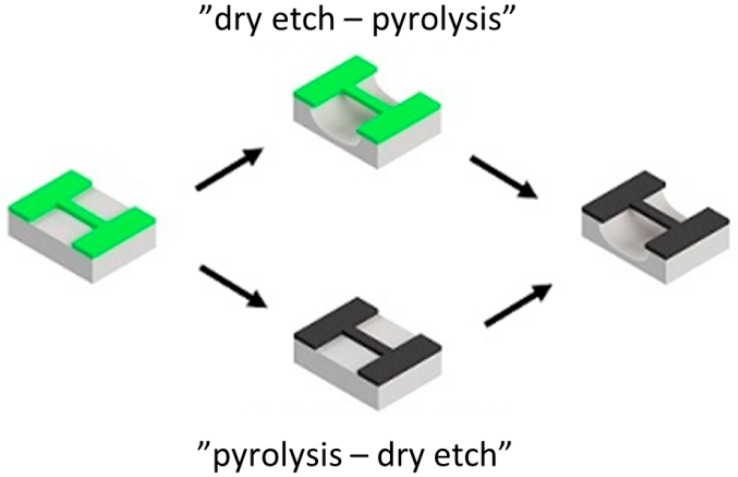
Two fabrication strategies for pyrolyzed photoresist microresonators. In the schematic grey is the Si substrate, green is the photoresist and black is the carbon.

The two fabrication strategies called “*dry etch-pyrolysis*” and “*pyrolysis-dry etch*” are schematically illustrated in Figure 1. In both cases, the photolithography was performed with positive AZ 5214e resist as well as negative epoxy SU-8 2005 resist.

Each process started with the spin-coating of a single layer of photoresist on the surface of 525-µm-thick 4 inch Si wafers. The wafers for AZ resist (AZ Electronic Materials, Somerville, NJ, USA) were dipped in BHF for 60 s to remove the native oxide. Spin-coating for 30 s with an acceleration of 1000 rpm/s and a spin speed of 725 rpm was performed to obtain a resist thickness of 4.2 µm. The photoresist was soft-baked at 90 °C for 60 s and patterned by UV exposure with a dose of 100 mJ/cm^2^. This was followed by development in AZ 351B diluted 1:5 in water for 70 s, rinse with water for 3 min and drying in the spin rinse dryer for 90 s. For the SU-8 resist (MicroChem, Westborough, MA, USA) a dehydration bake at 250 °C for 30 min was performed for the wafers prior to film deposition. Spin coating for 30 s with an acceleration of 5000 rpm/s and a spin speed of 2000 rpm resulted in a 5.5 µm thick SU-8 film. After spin-coating the wafers were placed in a ventilated area for 2 h to partially evaporate the solvent [19] followed by UV exposure with a dose of 200 mJ/cm^2^. The post exposure bake of the SU-8 was done on a hotplate with a temperature ramp of 2 °C/min up to 50 °C. After 60 min the hotplate was switched off and allowed to cool down to room temperature. The SU-8 was developed in propylene glycol methyl ether acetate (PGMEA) for 2 × 2 min, rinsed with 2-propanol (IPA) and dried in air for 60 min.

Releasing the microresonators by under-etching required highly isotropic etching of Si while avoiding damage of the structures of interest. Hence, dry etching using a Deep Reactive Ion Etcher (standard rate ASE, Surface Technology Systems, Newport, UK) was chosen. The plasma etching generates heat and radiation, which potentially cause high intrinsic stress and large deformations in the suspended structures and eventually affect the further usability of the devices. Therefore, a dry etch recipe earlier optimized for the release of thin SU-8 cantilevers was applied [20]. The process was conducted with a coil power of 1500 W, SF_6_ gas flow rate of 300 sccm to increase the chemical etching of Si and without use of O_2_ gas to minimize the etching of photoresist. The chuck temperature was kept at 0 °C to minimize thermal stress in the microresonators. In addition the platen was switched off to enhance the isotropic etch. The etch time should be kept as short as possible to minimize etching of the photoresist and prevent heating. Here, 5 min were enough to ensure that the majority of the structures were successfully released. Only the widest resonators, with a width of 50 µm, were still connected to the Si substrate after the process.

For pyrolysis, the wafers were loaded into a PEO-601 furnace (ATV Technologie GmbH, Vaterstetten, Germany) with a N_2_ gas flow rate of 24 L/min and first heated from room temperature to 200 °C. Maintaining this temperature for 30 min supports elimination of solvents from the photoresist and residual O_2_ from the furnace. Then the temperature was increased to 900 °C and kept constant for 60 min to complete the carbonization. Finally the oven was cooled down to room temperature. The ramp rate for all temperature changes was 2 °C/min.

### 2.2. Determination of Pyrolyzed Photoresist Density and Thickness

Unpatterned photoresist films were processed on 4 inch Si wafers with identical fabrication parameters as described above for photolithography and pyrolysis. The samples were weighed on a microbalance before spin-coating and after pyrolysis of the polymer films to determine the mass of the pyrolyzed photoresist. The carbon layer was mechanically removed at several points across the wafer and the film thickness was determined using a contact profilometer. The data was used to obtain the average thickness of the pyrolytic carbon. The uncertainties were evaluated based on the standard deviation of the mean assuming a t-distribution for a confidence interval of 95%. The film thickness was *h_SU8-C_* = 1.00 ± 0.02 µm for SU-8 based carbon (SU8-C) and *h_AZ-C_* = 550 ± 16 nm for pyrolyzed AZ 5214e (AZ-C) which is in good agreement with reported values of vertical shrinkage during pyrolysis [18,21]. By knowing the mass and the volume of pyrolyzed photoresists, the density was calculated to be *ρ_SU8-C_* = 1.52 ± 0.06 g/cm^3^ for pyrolyzed SU-8 and *ρ_AZ-C_* = 1.42 ± 0.06 g/cm^3^ for pyrolyzed AZ which is similar to values reported previously [11,12].

### 2.3. Resonance Frequency Measurements

The experiments were conducted at room temperature in high vacuum at a pressure below 10^−5^ mbar where air damping is negligible [22]. The chips were glued directly onto a piezoelectric actuator. The resonance frequency of the out-of-plane vibration was read-out by a laser Doppler vibrometer (MSA-500 from Polytec GmbH, Waldbronn, Germany). The laser power was kept at the minimum level at all times to minimize heating of the microstructures.

## 3. Results and Discussion

### 3.1. Fabrication with “Dry Etch-Pyrolysis” Process

**Figure 2:**
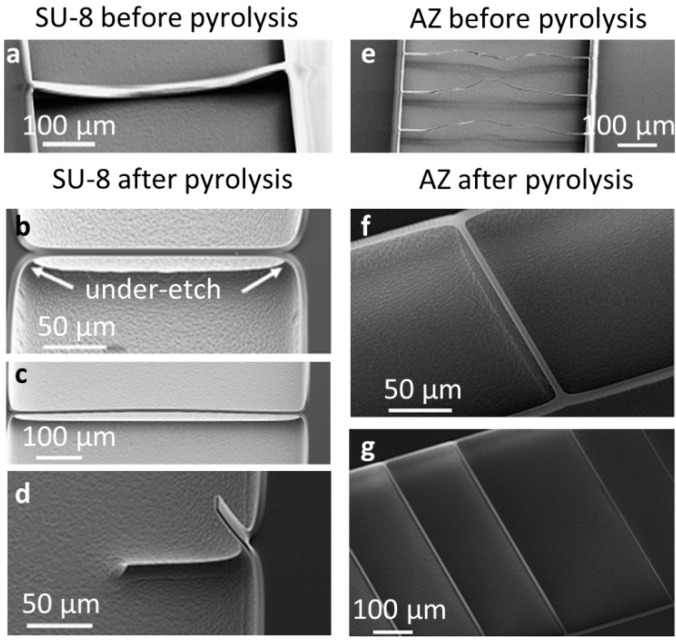
SEM pictures of the fabrication output of the “*dry etch-pyrolysis*” strategy. (**a**) SU-8 photoresist resonators (*L* = 500 µm, *w* = 14 µm) collapsed after dry etch; (**b**) Suspended SU8-C (*L* = 200 µm, *w* = 30 µm); and (**c**) (*L* = 500 µm, *w* = 30 µm) resonators; (**d**) SU8-C cantilever (*L* = 100 µm, *w* = 30 µm) with high initial bending; (**e**) AZ photoresist resonators (*L* = 500 µm, *w* = 6 µm) with severely distorted structure due to high local temperature during dry etch; (**f**) Short AZ-C (*L* = 200 µm, *w* = 14 µm); and long (**g**) (*L* = 500 µm, *w* = 14 µm (**left**) and *w* = 6 µm (**right**)) resonators. All dimensions are nominal mask dimensions.

Figure 2 summarizes the fabrication results for the “*dry etch-pyrolysis*” processing strategy. All cantilevers and doubly-clamped structures except the 50-µm-wide resonators were successfully released.

Figure 2a,e show doubly-clamped SU-8 and AZ strings after the dry etch but before pyrolysis. Although the dry etch recipe was optimized to reduce intrinsic and extrinsic stress in released polymer microstructures [20], doubly-clamped photoresist strings experienced structural changes during dry etching, which resulted in a contorted and undefined shape, in particular at increased distance from the clamping (see Figure 2e).

Figure 2b,c,f,g show doubly-clamped SU8-C and AZ-C strings. After pyrolysis, the deformed doubly-clamped resist strings were transformed into straight and well-defined suspended doubly-clamped pyrolytic carbon structures. This straightening was caused by a considerable shrinkage of the photoresist during pyrolysis. The isotropic Si dry etching resulted in an under-etch of 23 µm at the clamping of the beams. After pyrolysis, the width of the overhanging area decreased to around 11 µm, resulting in an elongation of the doubly-clamped resonators of around 25 µm independent of the initial length of the beams. Furthermore, the width of the resonators was also considerably reduced due to shrinkage. For example, suspended AZ resonators with a nominal width of *w* = 14 µm before pyrolysis had a final width of only around 6 µm. The narrowest doubly-clamped AZ strings with a nominal width of *w* = 3–6 µm and *L* > 500 µm ruptured. For doubly-clamped pyrolytic carbon resonators obtained from SU-8 resist, buckling was generally observed as presented in Figure 2c.

For both photoresist types, fabrication of singly-clamped beams with the “*dry etch-pyrolysis*” processing strategy was unsuccessful due to large initial bending of the microstructures after pyrolysis (see Figure 2d).

Finally, the comparison of the fabricated structures indicates a difference in behavior between SU-8 and AZ photoresist during pyrolysis (Figure 2b,f). The doubly-clamped resonators obtained with AZ as the precursor had a relatively well-defined clamping area with a sharp corner. In contrast, the structures based on SU8-C showed a smooth transition between string and clamping edge. This difference can be associated with different levels of lateral shrinkage and resist reflow of positive (AZ) and negative (SU-8) photoresists during pyrolysis [9]. The geometry of the clamping area of the SU8-C structures could contribute to increased radiation of vibrational energy into the substrate, which could increase support loss.

In conclusion, the “*dry etch-pyrolysis*” process based on the AZ resist precursor yielded doubly-clamped pyrolytic carbon string resonators with tensile stress.

### 3.2. Fabrication with “Pyrolysis-Dry Etch” Process

**Figure 3:**
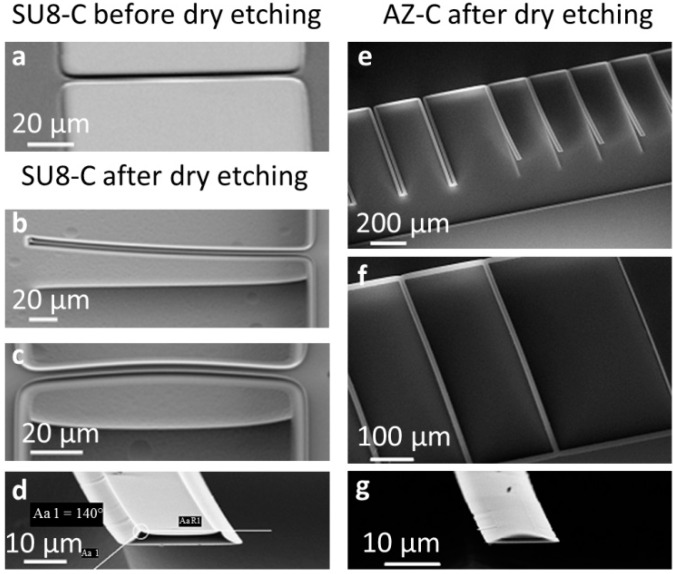
SEM pictures of the structures obtained with the “*pyrolysis-dry etch*” strategy. (**a**) Unreleased SU8-C resonator (*L* = 100 µm, *w* = 14 µm) after pyrolysis; (**b**) Released SU8-C cantilever (*L* = 200 µm, *w* = 14 µm); (**c**) Doubly-clamped SU8-C beam (*L* = 100 µm, *w* = 14 µm); (**d**) Curved sidewalls of the cross-sectional profile of SU-8 cantilevers (*w* = 14 µm); (**e**) The widest pyrolyzed AZ cantilevers (**left**, *L* = 500 µm, *w* = 50 µm) were not released compared to the more narrow ones (**right**, *L* = 500 µm, *w* = 30 µm); (**f**) AZ-C doubly-clamped beams (*L* = 500 µm, *w* = 14 µm (**left**) and *w* = 6 µm (**right**)); (**g**) AZ-C cantilever (100 µm long, 14 µm wide) with a rounded profile due to the reflow of the photoresist. All dimensions are nominal mask dimensions.

The fabrication results of the “*pyrolysis-dry etch*” process strategy are summarized in Figure 3.

Figure 3a shows a doubly-clamped SU-8 resist beam after pyrolysis but before dry etching. The carbon structures were well defined and no significant dimensional change was observed. Apparently, lateral shrinkage occurs mostly for features that are not in direct contact with the substrate such as the ones obtained with the “*dry etch-pyrolysis*” process described above.

Figure 3b–g show pyrolytic carbon microstructures after dry etching. All microstructures except resonators with a width of 50 µm (Figure 3e, left) were completely released during dry etching. The singly-clamped cantilever beams obtained with this process showed no deformation at the clamping and only slight curvature for both SU-8 and AZ resist precursors (Figure 3b,e), even for devices with a length of 600 µm. However, doubly-clamped beams were clearly buckling after dry etching for both SU8-C and AZ-C (Figure 3c,f).

Figure 3d,g illustrate the beam profiles at the free-standing tip of the cantilever beams of SU8-C and AZ-C, respectively. The curved sidewalls of the obtained resonators are a consequence of non-uniform lateral shrinkage of the photoresist during pyrolysis. Shrinkage at the bottom was smaller than at the top surface of the structures of interest because it was restrained by interaction with the substrate [23]. This was most evident for the SU-8 structures (Figure 3d) [24], whereas for AZ, apparently, the reflow of the photoresist was possible and the cross-sectional profiles of the beams were more rounded (Figure 3g) [9].

In conclusion, the “*pyrolysis-dry etch*” process based on SU-8 and AZ resist precursors yielded singly-clamped pyrolytic carbon cantilevers.

### 3.3. Resonant Behavior of Cantilevers

**Figure 4:**
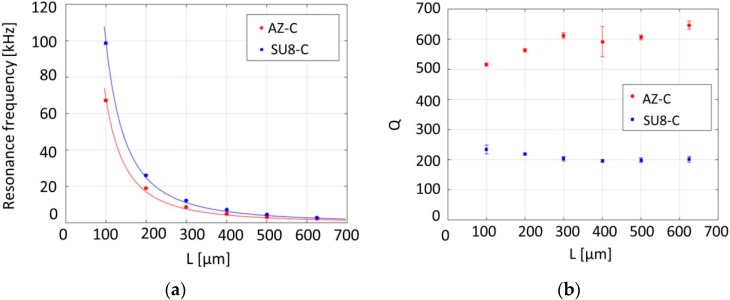
Relation between length and (**a**) resonance frequency of the fundamental bending mode and (**b**) Q of the carbon cantilevers fabricated with the “*pyrolysis-dry etch*” scheme using SU-8 and AZ photoresist precursors. Error bars represent a 95% confidence interval. Measurements were done on five cantilevers and then averaged.

The cantilevers fabricated with the “*pyrolysis-dry etch*” processing strategy with a nominal width of 30 µm, thicknesses of 1 µm and 550 nm for the SU-8 and AZ resists, respectively, and different lengths from 100 to 625 µm were used in these experiments. Figure 4 presents the measured resonance frequency and Q values as a function of the cantilever length.

The resonance frequencies are proportional to the inverse of the length squared, as given by the fundamental eigenfrequency of a singly-clamped Euler-Bernoulli beam with a rectangular cross-section

(1) $$\;f_{1}\approx \frac{1}{2\pi }\frac{h}{L^{2}}\sqrt{\frac{E}{\rho }}$$

with the Young’s modulus *E*.

The measured Q values show no significant dependence on the cantilever length, which suggests that intrinsic losses dominate over support losses. Indeed, with a wafer thickness of *h_sub_* = 525 µm, support loss can be estimated by *Q_support_*^−1^ ≅ (*w*/*L*) × (*h*^2^/*h_sub_*^2^) [25], which for the shortest cantilevers gives *Q_support_* ≅ 10^6^. This is four orders of magnitude higher than the measured Qs and hence support losses can be neglected. 

Pyrolytic carbon has a distorted lattice structure with graphite-like areas [21]. Therefore, the dominant energy loss that limits Q is believed to be a result of internal material damping, such as friction between carbon layers. According to the measurements, cantilevers fabricated with pyrolyzed AZ photoresist exhibited higher Qs than the ones based on SU-8.

Employing Equation (1) and the measured fundamental mode resonance frequency values, the Young’s modulus *E* was calculated (see Table 1). The Young’s modulus values are higher than values reported for pyrolytic carbon in the literature [11,12]. The results demonstrate that the properties of the pyrolytic carbon strongly depend on the type of precursor used and the pyrolysis conditions. The AZ-C shows a higher Young’s modulus than the SU8-C. This indicates that the AZ resist precursor results in pyrolytic carbon of higher quality, which also is reflected in the higher Qs measured for AZ-C (see Figure 4b). It should be noted that the non-rectangular cross-section along with the unknown residual stress distribution may to some extent influence the applicability of the simple singly-clamped beam model and eventually the calculation results. The calculated Young’s modulus values are more or less identical for cantilevers with a length *L* ≥ 300 µm. The values obtained for 100- and 200-µm-long cantilevers are lower due to the influence of the under-etched clamping, which is more important for shorter beams [26]. Therefore, data for the two shortest cantilevers was excluded from the calculation of the average values, which are 74 ± 8 GPa for SU8-C and 115 ± 8 GPa for AZ-C.

### 3.4. Resonant Behavior of Strings

**Figure 5:**
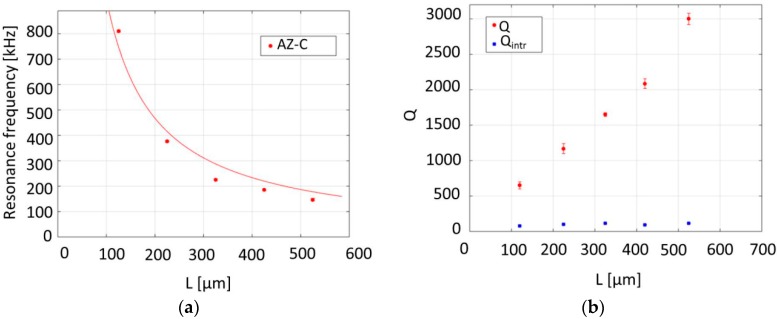
Relation between length, (**a**) resonance frequency (fit to Equation (2) included) and (**b**) Qs of the carbon strings fabricated with “*dry etch-pyrolysis*” strategy (**red**) and calculated values for resonators without tensile stress (**blue**). Error bars represent a 95% confidence interval.

Figure 5 shows the resonance frequency and Qs of AZ-C strings fabricated by the “*dry etch-pyrolysis*” process strategy as a function of length. The eigenfrequency of a string is given by

(2) $$f_{n}\;=\;\frac{n}{2L}\sqrt{\frac{\sigma }{\rho }}$$

where the tensile stress is σ, and the mode number is *n*.

A ratio between the fundamental resonance frequency mode (*n* = 1) and higher resonance modes of 2.02 ± 0.03 for the second harmonic (*n* = 2) to the fundamental mode and 3.1 ± 0.1 for the third harmonic (*n* = 3) to the fundamental mode were measured for 425-µm-long AZ-C string resonators, clearly confirming string-like behavior. The fundamental resonance frequency presented in Figure 5a was to a good approximation proportional to the inverse of the length, as predicted by Equation (2).

The measured resonance frequency values were used to calculate the tensile stress in the strings using Equation (2) and the results are listed in Table 2. Higher tensile stress was observed in shorter strings. This variation in stress could be the consequence of a different shrinkage of short strings during pyrolysis compared to longer ones. The resonance frequency values of 325-, 425- and 525-µm-long strings were fitted to Equation (2) and the calculated tensile stress was 33 ± 7 MPa.

In Figure 5b, the Q increases linearly with the string length, as predicted by the analytic model for damping dilution in strings [17]

(3) $$Q\approx \sqrt{3}\;\sqrt{\frac{\sigma }{E}}\frac{L}{h}Q_{intr}$$

with the intrinsic Q of an unstressed beam (e.g., a cantilever) *Q_intr_*.

*Q_intr_* of pyrolytic carbon strings was obtained (see blue data points in Figure 5b) by inserting the values of the Young’s modulus and tensile stress obtained from the experimental data (Table 1 and Table 2) in Equation (3). Here, we assume that it is a reasonable approximation to use the Young’s modulus values calculated using experimental data of structures fabricated with the “*pyrolysis-dry etch*” strategy. This resulted in an average value of *Q_intr_* for AZ-C resonators of *Q_intr_* = 100 ± 7. The intrinsic Q was relatively low and it was not significantly influenced by the string length. This suggests that *Q_intr_* is dominated by internal material friction. This value can directly be compared to the *Q_intr_* ≈ 600 obtained for the cantilevers (see Figure 4b). The material damping in the AZ-C cantilevers fabricated with the “*pyrolysis-dry etch*” process seems to be considerably lower than that of the AZ-C strings fabricated with the “*dry etch-pyrolysis*” process. This could suggest that the unpyrolyzed resist is affected more by the dry etch step compared to the pyrolytic carbon, finally resulting in carbon of lower quality.

## 4. Conclusions

The results of fabrication and characterization demonstrate that it is possible to obtain nanomechanical pyrolytic carbon cantilever and string resonators. The fabrication process consists of only three main steps: photolithography, dry etching and pyrolysis, which allows for shorter fabrication time in comparison with common methods based on Si or SiN resonators. The order of the two last process steps had a decisive influence on the fabrication output, as summarized in Table 3.

For the “*dry etch-pyrolysis*” process strategy, dry etching resulted in structural deformation of the photoresist and buckling of the doubly-clamped beams. However, during pyrolysis the lateral shrinkage of the released photoresist effectively induced tensile stress in the suspended doubly-clamped beams. Nevertheless, only pyrolyzed AZ 5214e strings could be used in further experiments and displayed a string-like behavior. Singly-clamped cantilever beams could not be used due to excessive bending in the clamping area.

For the “*pyrolysis-dry etch*” fabrication strategy, structures were first pyrolyzed and then released by dry etching. No change of the lateral dimensions was observed except for a change of the beam cross-section. The doubly-clamped structures were not stretched during pyrolysis and buckling was observed. As a consequence, these devices could not be used as string resonators. However, the singly-clamped cantilevers showed minimal bending and could be used for characterization of the Young’s modulus of pyrolytic carbon.

Quality factors of up to 3000 were obtained with pyrolytic carbon strings, which is one order of magnitude higher compared to the Qs obtained for the cantilevers. The relatively high Qs are the result of damping dilution due to intrinsic stress in the strings. With the obtained Qs and Young’s moduli of more than 100 GPa, pyrolytic carbon is a promising alternative material for the fabrication of micro- and nanomechanical resonators, allowing for a direct photoresist-based fabrication. Pyrolytic carbon has a density more than two times lower than SiN, which is an advantage in terms of, for instance, mass sensitivity. Even though SiN has substantially higher tensile stress [14], SiN string resonators would be just 50% more sensitive to attached mass. There is also plenty of room for improvement, especially by the optimization of pyrolysis conditions and structure design. Additionally, the intrinsic electrical conductivity allows for electrical integration for the development of novel carbon-MEMS/NEMS.

## Figures and Tables

**Table 1:** Values of Young’s modulus of cantilevers with different length. Errors represent a 95% confidence interval.

	SU8-C	AZ-C
L [µm]	E [GPa]	
100	58 ± 12	82 ± 11
200	65 ± 12	105 ± 15
300	72 ± 15	114 ± 16
400	77 ± 16	110 ± 15
500	74 ± 15	113 ± 16
625	74 ± 15	124 ± 17

**Table 2:** Values of tensile stress of strings with different lengths (nominal lengths defined in photolithography were 100, 200, 300, 400 and 500 µm). Error bars represent a 95% confidence interval.

AZ-C	
L [µm]	σ [MPa]
125	54 ± 5
225	41 ± 4
325	31 ± 3
425	35 ± 3
525	34 ± 5

**Table 3:** Summary of the overall fabrication output for the two different process strategies with the two precursor materials.

	Cantilevers		Strings	
Photoresist precursor	SU8-2005	AZ 5214e	SU8-2005	AZ 5214e
*dry etch-pyrolysis*	**×**	**×**	**×**	**√**
*Pyrolysis-dry etch*	**√**	**√**	**×**	**×**
